# Computational Analyses of an Evolutionary Arms Race between Mammalian Immunity Mediated by Immunoglobulin A and Its Subversion by Bacterial Pathogens

**DOI:** 10.1371/journal.pone.0073934

**Published:** 2013-09-03

**Authors:** Ana Pinheiro, Jenny M. Woof, Laurent Abi-Rached, Peter Parham, Pedro J. Esteves

**Affiliations:** 1 CIBIO Centro de Investigação em Biodiversidade e Recursos Genéticos/InBio Laboratório Associado, Universidade do Porto, Vairão, Portugal; 2 Departamento de Biologia, Faculdade de Ciências, Universidade do Porto, Porto, Portugal; 3 SaBio - Instituto de Investigación en Recursos Cinegéticos (CSIC-UCLM-JCCM), Ciudad Real, Spain; 4 Division of Cancer Research, Medical Research Institute, University of Dundee Medical School, Ninewells Hospital, Dundee, United Kingdom; 5 Centre National de la Recherche Scientifique, Laboratoire d’Analyse, Topologie, Probabilités - Unité Mixte de Recherche 7353, Equipe ATIP, Aix-Marseille Université, Marseille, France; 6 Department of Structural Biology, Stanford University School of Medicine, Stanford, California, United States of America; 7 CESPU, Instituto de Investigação e Formação Avançada em Ciências e Tecnologias da Saúde, Gandra PRD, Portugal; Wadsworth Center, New York State Dept. Health, United States of America

## Abstract

IgA is the predominant immunoglobulin isotype in mucosal tissues and external secretions, playing important roles both in defense against pathogens and in maintenance of commensal microbiota. Considering the complexity of its interactions with the surrounding environment, IgA is a likely target for diversifying or positive selection. To investigate this possibility, the action of natural selection on IgA was examined in depth with six different methods: CODEML from the PAML package and the SLAC, FEL, REL, MEME and FUBAR methods implemented in the Datamonkey webserver. In considering just primate IgA, these analyses show that diversifying selection targeted five positions of the Cα1 and Cα2 domains of IgA. Extending the analysis to include other mammals identified 18 positively selected sites: ten in Cα1, five in Cα2 and three in Cα3. All but one of these positions display variation in polarity and charge. Their structural locations suggest they indirectly influence the conformation of sites on IgA that are critical for interaction with host IgA receptors and also with proteins produced by mucosal pathogens that prevent their elimination by IgA-mediated effector mechanisms. Demonstrating the plasticity of IgA in the evolution of different groups of mammals, only two of the eighteen selected positions in all mammals are included in the five selected positions in primates. That IgA residues subject to positive selection impact sites targeted both by host receptors and subversive pathogen ligands highlights the evolutionary arms race playing out between mammals and pathogens, and further emphasizes the importance of IgA in protection against mucosal pathogens.

## Introduction

Immunoglobulin A (IgA), in the form of dimers or higher polymers (pIgA) particularly tetramers, is the predominant immunoglobulin isotype in mucosal tissues and external secretions, where it provides a major line of defense against pathogens. In addition, it plays a major role in the maintenance of the commensal microbiota in the intestinal tract, where interplay between commensal microorganisms and IgA promotes a mutually beneficial co-existence [1]. Monomeric IgA is present in serum, being the second most prevalent immunoglobulin after IgG and a critical factor for eliminating pathogens that breach external surfaces [2]. Much energy is expended in producing these serum and mucosal forms of IgA. In humans, for example, more IgA is produced than all the other antibody isotypes combined. Such high investment in IgA is presumably indicative of the key contribution this antibody isotype makes to immune protection. Like all immunoglobulins, IgA displays a basic monomeric structure of two light and two heavy chains, each having a variable and a constant region, linked together by disulphide bridges. Each chain is organized in globular domains consisting of approximately 110–130 amino acids. The light chains (VL and CL domains) and the variable (VH) and first constant domain of the heavy chain (Cα1) constitute the two Fab regions, which bind antigens. The remaining constant domains of the heavy chain (Cα2 and Cα3) constitute the Fc region, responsible for the recruitment of mechanisms that lead to pathogen elimination. Linking the Fab and Fc regions is a flexible hinge region. This basic IgA unit can exist as monomers or be arranged into dimers (dIgA) and higher order multimers in which the monomers are linked by a J (joining) chain. In secretions, IgA is present as secretory IgA (S-IgA), a complex of dIgA or pIgA with another polypeptide chain, the secretory component (SC) [3], which confers some protection from proteolytic cleavage.

IgA has been identified in all mammals and birds studied [3]. In mammals, differences in gene number and molecular forms have been noted, defining different IgA systems. Most mammals have one *IGHA* gene, coding for one IgA isotype, which adopts a dimeric form in serum IgA. Humans, chimpanzees, gorillas and gibbons have, however, two *IGHA* genes, which arose by gene duplication in a common hominoid primate ancestor and code for the IgA1 and IgA2 [4] subclasses. In hominoids serum IgA is mainly monomeric. Rabbit has the most complex IgA system observed, with 13 *IGHA* genes encoding 13 IgA subclasses [5]: of these 13 subclasses, 11 are expressed and are differentially distributed among the mucosal tissues [6]. Mammalian IgA subclasses mainly differ in the length and amino acid sequence of the hinge, which affects their susceptibility to cleavage by bacterial proteases [5], [7].

Elimination and destruction of pathogens is facilitated by the binding of Ig-antigen complexes to Ig receptors (FcRs) on effector cells and soluble effector molecules such as complement. In most mammals, IgA effector functions appear to be reliant on FcαRI (CD89), the Fc receptor specific for IgA: binding of the IgA-antigen complex to FcαRI can lead to phagocytosis, antibody dependent cell-mediated cytotoxicity (ADCC) and release of cytokines and inflammatory mediators. FcαRI binds to IgA at the Cα2–Cα3 interface [8], [9] an interaction that has been suggested to evolve under pressure from pathogen decoy IgA-binding proteins [10]. FcαRI appears to be functional in the majority of mammals, but it is notably absent from mice, rabbits and dogs due either to loss of the gene or to its degeneration into a pseudogene.

Other IgA-Fc receptors important for IgA function include the polymeric Ig receptor (pIgR) and the IgA/IgM Fc receptor (Fcα/µR) [11]. The pIgR is responsible for delivery of the large quantities of pIgA produced in the mucosae across the epithelial cell layer into mucosal secretions. In the process, pIgR is cleaved to yield the SC, which remains covalently complexed with pIgA to form S-IgA. The binding involves interaction of pIgR with J chain and IgA-Fc residues, particularly within the Cα3 domain of IgA. Some of these residues are located in the Cα2–Cα3 interface [12] and overlap with residues critical for binding to FcαRI and Fcα/µR [3]. In addition to transport of free pIgA, pIgR can also transport polymeric IgA immune complexes, including pIgA complexed with viruses, out across the epithelium [2]. Moreover, pIgA transported via pIgR may intercept and neutralize certain viruses inside epithelial cells [2]. In humans, Fcα/µR is present on macrophages and plasma cells, and also on follicular dendritic cells in tonsil and in intestinal tissues [11], likely reflecting a role in coordination of the immune response in mucosal tissues. The N-terminal Ig-binding domain of Fcα/µR shares similarity with domain 1 of pIgR, and the modes of interaction with dIgA are presumed to have similar features. Consistent with this possibility, the results of mutagenesis mapping analysis indicate a critical role for the Cα2–Cα3 domain interface of the IgA heavy chain in the interaction [13].

To evade elimination by the immune system, numerous pathogens have evolved proteins targeting IgA. These include IgA-binding proteins, which by binding to IgA block its access to host IgA-receptors, as well as proteases that by cleaving the IgA hinge, uncouple the recognition of foreign antigens from the effector functions that eliminate them. Examples of microbial IgA-binding proteins include the Sir22 and Arp4 proteins of *Streptococcus pyogenes*, the β protein of *Streptococcus agalactiae*, and the SSL7 toxin of *Staphylococcus aureus*, all of which bind to residues lying in the Cα2–Cα3 interface of IgA and prevent IgA interacting with FcαRI [14], [15]. Examples of the microbial proteases include IgA1 proteases secreted by clinically important bacterial pathogens, such as *Neisseria meningitidis* and *Haemophilus influenzae*, which cleave specifically in the hinge region of IgA1 of humans and great apes. IgA1 proteases are postproline endopeptidases that cleave at either Pro-Ser (type 1 enzymes) or Pro-Thr (type 2 enzymes) peptide bonds within the IgA1 hinge region. To achieve such specific cleavage, these enzymes recognize structural elements within the hinge [16], [17] and some of them also have to contact the Fc region before cleavage can occur [18], [19]. Notably, the type 2 IgA1 protease of *Neisseria meningitidis*, a causative agent of bacterial meningitis, interacts with the Cα3 residues of the Cα2–Cα3 interface also bound by FcαRI, pIgR and Fcα/µR, whereas the type 2 IgA1 protease of *Haemophilus influenzae* contacts a different set of Cα3 residues that are implicated in binding to pIgR [19].

Over recent years it has become increasingly apparent that S-IgA contributes to mucosal homeostasis through various mechanisms [20]. For example, coating of commensal bacteria by S-IgA may promote gut colonization and survival through biofilm formation. The role of S-IgA in maintaining the commensal microbiota may depend, at least in part, on interactions between IgA glycans and commensal bacteria [20].

Considering the complex interactions of IgA with other components of the immune system, with commensal microorganisms and the evasion proteins of diverse pathogens, IgA is a likely target for natural selection. Few studies have examined Ig sequences for the impact of natural selection and they have focused on IgA or IgG isotype in a limited number of vertebrate taxa [10], [21], [22]: for example, Abi-Rached et al [10] investigated the pattern of diversification of IgA-Fc using maximum likelihood [23], [24] and pairwise methods, with a focus on primates. To develop deeper understanding of the issue, in this study we took a broader approach that encompasses a wider range of methods and mammalian species. In total, 64 sequences from 28 species representing monotremes, marsupials and eight orders of placental mammals were included in the analyses.

## Methods

### Primate and Mammalian IgA Sequences

The complete sequences for primate IgAs used in a previous study [10] were obtained from GenBank (http://www.ncbi.nlm.nih.gov/genbank/); accession numbers are: Human IgA1 and IgA2 - J00220, J00221, M60192 and AJ012264; Chimpanzee IgA1 and IgA2 - X53702 and X53706; Gorilla IgA1 and IgA2 - X53703 and X53707; Gibbon IgA1 and IgA2 - X53708 and X53709; Orangutan IgA -X53704; Rhesus macaque IgA - AY039245 to AY039252, AY294614 and AY294615; Crab-eating macaque IgA - X53705 and Sooty mangabey IgA - AY544580 and AY544581.

Complete sequences for non-primate mammalian IgAs were obtained from IMGT (http://www.imgt.org/IMGTrepertoire/), GenBank (http://www.ncbi.nlm.nih.gov/genbank/) and Ensembl (http://www.ensembl.org/index.htm). In total 64 sequences from 28 species were included in the analyses, representing marsupials, monotremes, and eight orders of placental mammals: primates, artiodactyls, perissodactyls, rodents, carnivores, lagomorphs, chiropters and cetaceans. Accession numbers for the non-primate sequences used are: Cattle IgA - AF109167; Sheep IgA - AF024645; Pig IgA - U12594; Horse IgA - AY247966; Alpaca IgA - AM773729; Mouse IgA - J00475, AF175973 to AF175975, AH011154 to AH011156, and AY045750 to AY045752; Rat - ENSRNOT00000006888 and AY158661; Dog IgA - L36871; Panda IgA - AY818387; Rabbit IgA1 to 13 - X51647, X82108 to X82119; Big brown bat IgA - HM134938; Little brown bat IgA - HM134924; Short-nosed fruit bat IgA - HM134948; Black flying fox IgA - GQ427150; Dolphin IgA - AY621035; Possum IgA - AF091139 and AF027382; Opossum IgA - AF108225 and AF012110; Tasmanian devil - AFEY01402156; Platypus IgA1 and IgA2 - AY055778 and AY055779; Echidna IgA - AF416951. Excluded from the analysis was the recombinant human IgA2(n) allele [25] and the mouse IgA*3 allele for which the sequence has a nucleotide deletion in Cα1 that is presumably a sequencing or typographical error. The monotreme and marsupial Cα1 sequences were not included because of uncertainty in their alignment with the Cα1 domain sequences of placental mammals and also to avoid the risk of saturation that could result from including these highly divergent sequences; likewise, sequences of the rapidly-evolving IgA hinge region were excluded from the analysis.

For the analysis of the primate datasets and of the placental mammal Cα1 dataset, sequences were aligned using CLUSTAL W [27] as implemented in BioEdit [28], and corrected manually; notably, adjustments were made to follow the rigorous IMGT numbering system. For the mammalian Cα2 and Cα3 datasets, amino acid alignments were first generated using MUSCLE [29] and manual corrections, and these alignments were then used as a guide to prepare codon alignments for the same set of sequences.

Codon numbering is according to the Bur IgA1 numbering. IMGT unique numbering for C-DOMAIN [26] is also shown in parenthesis.

### Codon-based Analyses of Positive Diversifying Selection

To investigate positive selection on IgA, we studied the three constant domains (Cα1, Cα2 and Cα3) separately: for each domain we compared the rate per-site of nonsynonymous substitution (dN) to the rate per-site of synonymous substitutions (dS) in a maximum likelihood (ML) framework, using six different methods. Since each method has strengths and weaknesses, we used the approach of Wlasiuk and Nachman [30] to identify the codons for which the signal of positive selection was strongest: only codons identified by at least two of the ML methods were considered to be positively selected codons (PSC). Unlike pairwise dN/dS analyses, the methods used here rely on phylogenetic approaches and are thus not as sensitive as the pairwise dN/dS methods to differences in the number of sequences present in the taxonomic groups investigated: to increase the resolution of the analysis, we included all available sequences.

We first compared two alternative models implemented in CODEML (PAML 4.4) [23], [24]: M8, which allows for codons to evolve under positive selection (dN/dS>1) and M7, which does not (dN/dS≤1). These two nested models were compared using a likelihood ratio test (LRT) with 2 degrees of freedom [31], [32]. The analysis was run twice, and conducted with the F3×4 model of codon frequencies. Codons under positive selection for model M8 were identified using a Bayes Empirical Bayes approach (BEB) [33] and considering a posterior probability of >90%. For each analysis, a Neighbour-Joining phylogenetic tree was used as the ‘working topology’, and generated using Mega 5 [34] with the p-distance substitution model and the complete deletion option to handle gaps and missing data. Overall, the tree topologies used reflected the accepted topology for mammals.

We also used the five methods for detecting positive selection available from the DATAMONKEY web server [35]: the Single Likelihood Ancestor Counting model (SLAC), the Fixed Effect Likelihood model (FEL), the Random Effect Likelihood model (REL), the Mixed Effects Model of Evolution (MEME) and the Fast Unbiased Bayesian Approximation (FUBAR). For these analyses, the best fitting nucleotide substitution model was determined through the automatic model selection tool available on the server.

Because recombination can contribute to false inference of positive selection, causing a high rate of false positive detection [36], [37], [38], all datasets were screened for recombination using the GARD [39] method from the DATAMONKEY web server [35]. No evidence of recombination was found.

### Location of the PSC in Structural Models of IgA

A molecular model of human IgA1 (MMDB ID: 10546, PDB ID:1iga [40]) and the three-dimensional X-ray crystal structure of human IgA1-Fc (PDB ID :1OW0 [9]) were used to map the amino acids encoded by PSC onto 3D structures of the protein. To investigate their relation to putative sites of interest, the sites of interaction with host receptors (FcαRI, pIgR and Fcα/µR [3], [8], [9], [12], [13]) and bacterial proteins (*S. aureus* SSL7 protein, streptococcal IgA binding proteins, *N. meningiditis* and *H. influenzae* type 2 IgA1 proteases [3], [14], [19], [41]) were also mapped onto the 3D structure. For this purpose the NCBI application Cn3D 4.1 (http://www.ncbi.nlm.nih.gov/Structure/CN3D/cn3d.shtml [42]) and iMol software [43] were used. Although the molecular model of human IgA1 has the drawbacks of being based on low resolution X-ray and neutron scattering data and of using the X-ray crystal structure of IgG to model the Fc part of Iga (the IgA Fc structure was unavailable at the time), it offered the best means to visualize all PSC in one intact structure. The solved X-ray crystal structure of human IgA1-Fc offers a higher resolution view, and aids understanding of the putative impact of these PSCs on the IgA-Fc ligand interaction.

## Results

### Natural Selection Diversified the Cα1 and Cα2 Domains of Primate IgA Sequences

Using the ML approach of PAML [23], [24], evidence for positive diversifying selection was obtained in primates for two of the three IgA constant domains, Cα1 and Cα2, with the model allowing sites to evolve under positive selection (M8) showing a significantly better fit than the model that did not (M7) (α = 0.01–0.05; Table 1). The other five ML methods also identified positively selected sites for IgA Cα1 and Cα2 but not for IgA Cα3. Comparison of the sites characterised by each method reveals five codons supported with high confidence (p>0.9) by at least two methods: of these five positively selected codons (PSC), two are in the Cα1 domain, codons 133 and 166 (Cα1–10 and 45.2), and three others are in the Cα2 domain, codons 296, 319 and 326 (Cα2–84, 100 and 107). Natural amino acid variability and characteristics for each of these codons are given in Table 2: for four of the five positions (133, 166, 319, and 326), changes in amino acid characteristics such as polarity and charge were observed, with potential to alter the protein structure or capacity for protein–protein interaction.

**Table 1:** Phylogenetic tests of positive selection in primates.

	Test of selection				Sites under selection identified by different methods^a^							
IgA dataset	lnL M7(neutral)/lnLM8 (selection)	−2lnΔLc	Significance	p_s_, ω_s_ b	PAML M8 (p>0.5;Abi-Rachedet al, 2007)	PAML M8 (p>0.9;present study)	SLAC	FEL	REL	MEME	FUBAR	PSC^c^
CH1	**−**1212.4/−1200.0	24.8	** (p<0,01)	0.03, 3.86	n.a.	133, 166, 197	none	none	133, 137, 166	133, 136,_138, 165	133	133, 166
CH2	**−**1365.3/−1361.3	8.0	* (p<0,05)	0,02, 2.6	245, 296, 317, 319, 326, 331, 333	319, 326	none	245, 296, 319	273, 293, 296, 317	296, 324, 326, 330	319	296, 319, 326
CH3	**−**907.08/−907.08	0.0	n.s.	0.00, 1.0	none	none	none	none	None	none	none	–

a Codons identified by more than one ML method are underlined.

b ps = proportion of the sites under selection, ωs = estimated dN/dS of the sites under selection in M8.

c Positively Selected Codons: only the codons identified by at least two of the ML methods were considered to be positively selected codons.

**Table 2:** Characterization of natural amino acid variation for each residue identified under positive selection in primate IgA.

			Natural amino acid variation			
	Residue^a^	Functional information	H, +^b^	H, - b	H, n b	HY, n b
**Primates Cα1**						
	133	Close to hinge	K	D, E	–	C
	166	Exposed	R, K, H	–	S, Q, T	P, I, L
**Primates Cα2**						
	296	Exposed	R, H	–	–	–
	319	Exposed; close to SSL7 binding site	K	E	Q	V
	326	Exposed	–	E	N	A

a IgA1 Bur numbering.

b amino acid characteristics: H- Hydrophilic, HY- Hydrophobic, +- positive, –negative, n-neutral.

### Mammalian IgA Evolution was Marked by Diversifying Selection on the Three Constant Domains

**Figure 1:**
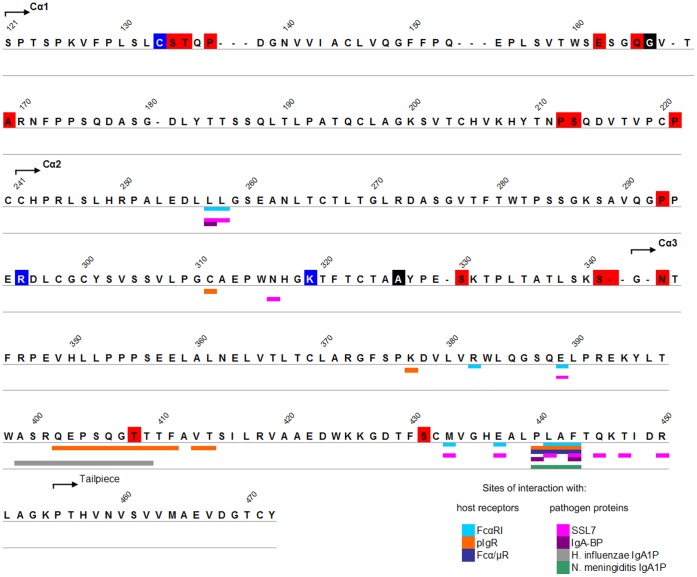
Residues encoded by PSC in human IgA1 Cα1, Cα2 and Cα3 domains. Residues encoded by PSC are highlighted in red (selected in mammals), black (selected in primates and mammals) or blue (selected in primates). Sites of interaction with studied ligands are indicated below the sequence according to the colour panel on the figure. Bur numbering is shown above.

Analysis of IgAs from a much broader range and larger sample of mammals using the ML approach implemented in PAML [23], [24] revealed significant evidence of diversifying selection for two of the three domains investigated (Cα1 and Cα2) (Table 3). However, the other five ML methods clearly identified positively selected sites for all three domains, which is consistent with the three domains having been the targets of diversifying selection. Comparison of the positively selected sites identified by each of the methods led to the identification of eighteen well supported positively selected codons (PSC) (Table 3). Of these eighteen PSC, ten locate to the Cα1 domain, five to the Cα2 domain, and three to the Cα3 domain (Figure 1). All but one of these residues show variations in both polarity and charge, changes that could alter the protein structure or capacity for protein–protein interaction (Table 4); the exception, PSC 431 (Cα3–103), displays a restricted set of residues that share the same polarity and charge, suggesting these characteristics are of value at this position. Of note, two of the changes at PSC in the Cα1 domain, codons 166 and 213 (Cα1–45.2 and 116 IMGT numbering) can generate putative N-glycosylation sites, which could affect protein function: residue 166 is a known N-glycosylation site of primate IgA2, as well as sheep, panda and alpaca IgA and rabbit IgA7, IgA8, IgA11 and IgA13. In contrast the putative N-glycosylation site at residue 213 appears only in rabbit IgA7, IgA8, IgA11 and IgA13.

**Table 3:** Phylogenetic tests of positive selection in mammals.

	Test of selection				Sites under selection identified by different methods^a^						
Dataset	lnL M7(neutral)/lnLM8 (selection)	−2lnΔLc	Significance	p_s_, ω_s_ b	PAML M8	SLAC	FEL	REL	MEME	FUBAR	PSC^c^
Placental mammalsIgACH1	**−**6035.6/−6010.7	49.8	** (p<0,01)	0.19, 1.79	121, 126, 134, 135, 136, 137, 138, 139, 157, 162, 166, 170, 194, 198, 199, 200, 201, 217, 221	162, 165, 169, 212, 213	135, 162, 165, 169, 212, 213, 221	221	124, 133, 134, 135, 137, 148, 162, 166, 169, 212, 213, 219, 221	135, 169	**134, 135, 137, 162, 165, 166, 169, 212, 213, 221**
Mammals IgACH2	**−**6075.9/−6068.9	14	** (p<0,01)	0.06, 1.34	243, 282, 293, 317, 330, 341a	330	341, 341a	284, 292, 293, 326, 341, 341a	242, 264, 285, 294, 326, 330, 337, 340, 341, 341a	341a	**293, 326, 330, 341, 341a**
Mammals IgACH3	**−**5674.91/−5672.44	4.93	n.s. (p<0,1)	0.00, 1.00	none	343, 364, 408	343	431	343, 389, 394, 408, 423, 427, 431, 442, 451	343	**343, 408, 431**

a Codons identified by more than one ML method are underlined.

b ps = proportion of the sites under selection, ωs = estimated dN/dS of the sites under selection in M8.

c Positively Selected Codons: only the codons identified by at least two of the ML methods were considered to be positively selected codons.

**Table 4:** Characterization of natural amino acid variation for each residue identified under positive selection in mammal IgA.

			Natural amino acid variation			
Residue^a^		Functional Information	H, +^b^	H, -b	H, n^b^	HY, n^b^
**Placental mammal Cα1**						
	134	Close to hinge	H, R, K	–	N, Q, S, C, Y	P, L, I, G
	135	Close to hinge	H, R	E, D	T, N, Q, S, C	P, V, A, L, I, G
	137	Close to hinge	–	D	Q, S, C	P, L, A
	162	Exposed	K, H	E, D	Q, T, S	A, P, I, V, G, L
	165	Exposed	K	E, D	Q, S	A, P
	166	Exposed	–	D	N, S	G, V
	169	Exposed	–	D	N, T, S	A, V, I, G
	212	Orientated towards variable domains, exposed	K	E	T, S	P, A, V, I
	213	Orientated towards variable domains, exposed	R, H	D	N, S	V, G, I, L
	221	Bordering hinge	R, K, H	–	S, Q, T	P, I, L
**Mammals Cα2**						
	293	Quite close to hinge, exposed	R, K	E	N, T, S	P, A, L
	326	Exposed	K	E	N, T, S	A
	330	Exposed	K	E,D	T, S	L, V, A, F
	341	Positioned on strand linking Cα2 and Cα3; vicinity of SSL7 binding site	–	D	T, S	P, V, A, L, I
	341a	Positioned on strand linking Cα2 and Cα3; vicinity of SSL7 binding site	K, R	–	T, S	L
**Mammals Cα3**						
	343	Positioned on strand linking Cα2 and Cα3; vicinity of SSL7,pIgR, and *H.influenzae* IgA1P binding site	–	E, D	N, T, S	P, V, A, I
	408	pIgR, and *H.influenzae* IgA1P binding site	K	–	T, S	I, P, A, V, G
	431	Positioned in middle of β-strand, exposed	–	–	N, T, S	–

a IgA1 Bur numbering.

b amino acid characteristics: H- Hydrophilic, HY- Hydrophobic, +- positive, –negative, n-neutral.

The recently developed MEME methodology [44] can identify both episodic and persistent positive selection, because it allows the distribution of the dN/dS ratio to vary from site to site and also from branch to branch at a site. The additional positively-selected codons identified by MEME and not by the other approaches, are likely to have been subject to episodes of positive selection. Consistent with this interpretation, of 6 such sites detected by MEME in the Cα3 domain residues 389 and 442 are sites targeted by pathogenic IgA-binding proteins (Cα3– 45.2 and 115 IMGT numbering).

### Positively Selected Codons are Located Near Sites of Interaction with Ligands and Bacterial Proteases

**Figure 2:**
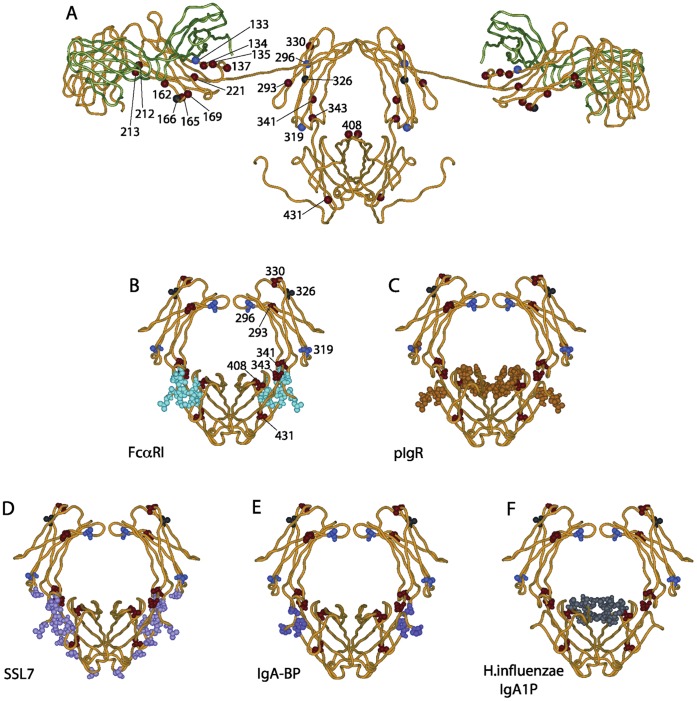
Residues encoded by PSC in the three-dimensional structure of human IgA1 and their relationship to sites of biological interest. A, Model of human IgA1 (PBD ID 1iga) with residues encoded by PSC highlighted. The light chains are colored green and the heavy chains colored yellow. Positively selected positions are represented by red dots (selected in mammals), black dots (selected in primates and mammals) or blue dots (selected in primates). B to F, Human IgA1-Fc (PDB ID 1OW0) with residues critical for FcαRI interaction (B), pIgR interaction (C), SSL7 interaction (D), streptococci IgA binding proteins interaction (E), and *H. influenzae* type 2 IgA1 protease interaction (F) highlighted in cyan, orange, pink, purple and grey respectively. Positively selected positions are represented as in panel A. Differences between the Fc structures in A and B-F reflect the fact that the model of intact IgA1 in A is based on low resolution X-ray and neutron scattering modeled based on the X-ray crystal structure of IgG Fc (the closest structure available at the time of modeling), while B-F show an X-ray crystal structure of human IgA1-Fc solved as part of a complex with FcαRI (not shown).

To understand better the possible biological significance of the detected PSC, we mapped the residues they encode onto a molecular model of human IgA1 and the three-dimensional X-ray crystal structure of IgA1-Fc, along with sites of interaction for host receptors and bacterial proteins (Figure 2). Remarkably, more than half (13 out of 21) of the PSC encode residues located near sites of interaction with ligands and bacterial proteases. Cα1 residues 133, 134, 135, 137 and 221 and Cα2 residues 293 and 296 (Cα1–10, 11, 12, 14 and 124 and Cα2–81 and 84, IMGT numbering) are near the hinge region, the preferential target region for some IgA1-specific bacterial proteases. Cα1 residues 212 and 213 (Cα1–115 and 116) have a general orientation towards the variable domains involved in antigen recognition. Cα2 residues 341 and 341a (Cα2–124 and 125) and Cα3 residue 343 (Cα3–1.3) are part of the exposed strand linking the Cα2 and Cα3 domains of IgA1, in the vicinity of the Cα2 NH motif that participates in the binding of *S. aureus* SSL7 molecules to human IgA [25]. Residue 343 also lies close to the putative interaction site for pIgR [12] and a region important for interaction with the type 2 IgA1 protease of *H. influenzae* [19]. Residue 408 (Cα3–85.5) is one of several Cα3 domain residues of human IgA1 that directly influence binding to pIgR; it also lies adjacent to the site where the *H. influenzae* IgA1 protease is believed to bind. Although position 431 (Cα3–103) in the Cα3 domain is positively selected, its location in the IgA molecule is not close to any known interaction sites of IgA-Fc region. Substitutions at this position could exert a functional effect by indirectly influencing the conformation of one or more of the interaction sites.

## Discussion

Genes involved in host-pathogen interactions are prone to diversifying selection [45], [46]. As pathogens continuously evolve mechanisms to evade host defenses and cause infectious diseases, so must host species evolve counter defense mechanisms if they are to survive. This never-ending arms race subjects those components of the mammalian immune system that recognize pathogens and their products to strong varying selection. IgA, the main Ig isotype present in external secretions and at mucosal surfaces, is uniquely exposed to a wide variety of bacteria, viruses, fungi and other infectious microorganisms, which together exert strong selective pressures on this immunoglobulin isotype. The results obtained in this study demonstrate the considerable impact that positive selection has played in the evolution of IgA in mammals and in the diversity and divergence of IgA among extant mammalian species.

### Natural Selection Diversified IgA in Mammals

Consistent with the study of Abi-Rached and coworkers [10], our analysis shows that the Cα2 domain of primate IgA-Fc exhibits evidence of positive diversifying selection, and the Cα3 domain does not. Making use of six different and complementary methodologies to identify positively selected residues, three Cα2 codons were identified by at least two of the methods used (positions 296, 319 and 326). These three positions also correspond to three of the seven codons identified previously [10] as being positively selected (Table 1). In contrast, the other four positions found previously as positively selected did not reach the cutoffs for detection used here, even though two of them appeared in individual analyses (positions 245 and 317, Table 1). Because the goals of the two studies were different (sensitive detection in the earlier study versus detection of positions with the strongest signals for selection here), different cutoffs were applied. To reconcile the apparent discrepancies will require analysis of a much larger dataset of IgA sequences.

To develop deeper understanding of IgA evolution, we compared IgA in a broad range of mammalian species. Of eighteen positions selected during mammalian evolution, only two are included in the five positions selected during primate evolution. This difference vividly illustrates the evolutionary plasticity of IgA.

We find that diversifying selection has mainly targeted the Cα1 and Cα2 domains of IgA, and to lesser extent the Cα3 domain. Thus only three of the eighteen selected positions are in the Cα3 domain. One of these, position 431, exhibits relatively conservative variation, having only three alternative amino acids, with similar polarity and charge. The Cα3 domain, along with the J chain, plays a key role in binding of pIgA to pIgR. Cα3 is also the main domain of IgA involved in binding to the major IgA-Fc receptors FcαRI and Fcα/µR. These crucial roles, along with contributions to the assembly and polymerization of IgA, can explain why Cα3 is the most conserved of the constant region of the IgA heavy chain. In contrast, the ten PSCs detected in Cα1 show more variety in amino acid substitutions, including changes in polarity and charge. Such variation modulates the Cα1 structure, with potential impact on Fab conformation, the antigen-binding site and the hinge region. Substitution at residues 166 and 213 could introduce an additional N-glycosylation site since this putative site of glycosylation is also present in primate IgA2, sheep, panda and alpaca IgA, and some rabbit IgA subclasses. N-linked glycans in the Fab region are known to influence antigen binding, either by increasing affinity for antigen or blocking antigen binding [47]. Since IgA-Fc N-linked glycans could protect IgA from cleavage by bacterial and other proteases [18], we speculate that Fab N-linked glycans can also contribute to such protection from proteases. Furthermore, glycans could impact on interactions of S-IgA with commensal microorganisms, thereby influencing the make-up of the microbiota and homeostasis of the gut [1], [20].

### IgA Diversification in Mammals Targets Sites Involved in the Interaction with Ligands and Bacterial Proteases

Mapping positively selected sites onto the structures of IgA and IgA-Fc revealed their likely impact on IgA function. Seven such sites, residues 133, 134, 135, 137 and 221 and Cα2 residues 293 and 296, are near the hinge, which links the antigen-recognition function of the Fab arms to the effector-recognition function of the Fc region. Because it is accessible, flexible and essential for antibody function, the hinge is a preferred target for bacterial proteases [3], [48]. Hinge structure varies considerably across mammalian species and between different subclasses and allotypes. For example, the hinge of hominoid IgA1 is 16 amino-acids longer than that of IgA2 and much more susceptible to proteolytic cleavage. The possible advantage of the longer hinge in IgA1 is its greater flexibility and potential for cross-linking antigens on the surface of bacteria and other pathogens [2]. Longer hinges are also a feature of most rabbit IgA subclasses. Thus, any variation that confers protection of the IgA hinge from proteolysis is a likely candidate for positive selection. For IgA1 proteases that cleave specifically in the hinge of hominoid IgA1 the distance of the susceptible peptide bond in the hinge from the “top” of the Fc (where the heavy chain enters the globular Cα2 domain) is critical for efficient cleavage [17]. Indeed, the crystal structure of a bacterial IgA1 protease from *H. influenzae* suggests that an intricate and coordinated association of protease with IgA is essential for optimal orientation of the hinge into the enzyme’s active site [49]. Substitutions at residues in and around the hinge could therefore increase resistance to proteolytic attack and become targets for positive selection.

Three positively-selected residues are found near sites of human IgA1 that interact with Fc receptors and bacterial proteins. Residues 341, 341a and 343 are in the strand linking the Cα2 and Cα3 domains, in the vicinity of the Cα2 asparagine-histidine motif that participates in the binding of *S. aureus* SSL7 molecule to human IgA [25]. The CH2–CH3 interface is central to the binding of IgA to several classes of Fc receptor including FcαRI, Fcα/µR and pIgR [8], [9], [12], [13], and is also the target of pathogenic mechanisms to obstruct IgA function [14], [15], [19]. Variation at the CH2–CH3 interface could prove adaptive, either by improving the binding of IgA to its Fc receptors or hampering the binding of pathogen decoy molecules, or by achieving both of these effects. Such adaptations could be accomplished by changes in the residues that contact Fc receptors or decoy proteins and also in nearby residues that have conformational impact. Residues under positive selection have been described in the Cγ2–Cγ3 interface of IgG in leporids [21]. Residue 408, is one of the positively selected Cα3 residues implicated in the binding of human IgA1 to pIgR [12] and the type 2 IgA1 protease of *H. influenzae*. Substitution at position 408 could therefore provide protection from cleavage by this IgA protease. The results of mutagenesis experiments are consistent with this possibility [19].

MEME methodology, which detects both episodic and persistent positive selection, identified codons in all three IgA analysed domains that were not revealed by the methods detecting only persistent selection. Thus these PSC are candidates for being subject to episodic selection. Among them are residues 389 and 442 in Cα3 that are targets for pathogenic IgA-binding proteins. Residue 442, which was previously shown to be subject to episodes of diversifying selection [10], is a site of N-linked oligosaccharide for IgA in mice. The glycan attached at asparagine 442 of mouse IgA hinders interaction with the *S. aureus* SSL7 decoy protein, but does not affect the binding of IgA to pIgR [50].

In conclusion, this study identified residues under positive selection in all three IgA heavy chain constant region domains. The majority of the identified residues are located in parts of the molecule that are essential for the functions of IgA in resistance to pathogens. This correlation is consistent with the positively-selected residues having influences on the interactions of IgA with immune-system receptors and the microbial proteins that interfere with these interactions. Future functional analyses should determine the mechanisms by which the positively selected residues exert their effect. Such knowledge could assist the design of therapeutic IgA-based monoclonal antibodies that are not susceptible to the pathogenic proteins that obstruct the defense functions of IgA.

## References

[1] Slack E, Balmer ML, Fritz JH, Hapfelmeier S (2012) Functional flexibility of intestinal IgA – broadening the fine line. Front Immunol 3: 100. 22563329 10.3389/fimmu.2012.00100 PMC3342566

[2] Woof JM, Kerr MA (2006) The function of immunoglobulin A in immunity. J Pathol 208: 270–282. 16362985 10.1002/path.1877

[3] Woof JM, Russell MW (2011) Structure and function relationships in IgA. Mucosal Immunol 4: 590–597. 21937984 10.1038/mi.2011.39

[4] Kawamura S, Saitou N, Ueda S (1992) Concerted evolution of the primate immunoglobulin alpha-gene through gene conversion. J Biol Chem 267: 7359–7367. 1559979

[5] Burnett RC, Hanly WC, Zhai SK, Knight KL (1989) The IgA heavy-chain gene family in rabbit: cloning and sequence analysis of 13 C alpha genes. EMBO J 8: 4041–4047. 2512120 10.1002/j.1460-2075.1989.tb08587.x PMC401579

[6] Spieker-Polet H, Yam PC, Knight KL (1993) Differential expression of 13 IgA-heavy chain genes in rabbit lymphoid tissues. J Immunol 150: 5457–5465. 8515070

[7] Schroeder HW Jr, Cavacini L (2010) Structure and function of immunoglobulins. J Allergy Clin Immunol 125: S41–52. 20176268 10.1016/j.jaci.2009.09.046 PMC3670108

[8] Pleass RJ, Dunlop JI, Anderson CM, Woof JM (1999) Identification of residues in the CH2/CH3 domain interface of IgA essential for interaction with the human fcalpha receptor (FcalphaR) CD89. J Biol Chem 274: 23508–23514. 10438530 10.1074/jbc.274.33.23508

[9] Herr AB, Ballister ER, Bjorkman PJ (2003) Insights into IgA-mediated immune responses from the crystal structures of human FcalphaRI and its complex with IgA1-Fc. Nature 423: 614–620. 12768205 10.1038/nature01685

[10] Abi-Rached L, Dorighi K, Norman PJ, Yawata M, Parham P (2007) Episodes of natural selection shaped the interactions of IgA-Fc with FcalphaRI and bacterial decoy proteins. J Immunol 178: 7943–7954. 17548632 10.4049/jimmunol.178.12.7943

[11] Monteiro RC, Van De Winkel JG (2003) IgA Fc receptors. Annu Rev Immunol 21: 177–204. 12524384 10.1146/annurev.immunol.21.120601.141011

[12] Lewis MJ, Pleass RJ, Batten MR, Atkin JD, Woof JM (2005) Structural requirements for the interaction of human IgA with the human polymeric Ig receptor. J Immunol 175: 6694–6701. 16272325 10.4049/jimmunol.175.10.6694

[13] Ghumra A, Shi J, McIntosh RS, Rasmussen IB, Braathen R, et al (2009) Structural requirements for the interaction of human IgM and IgA with the human Fcalpha/mu receptor. Eur J Immunol 39: 1147–1156. 19266484 10.1002/eji.200839184 PMC3118421

[14] Pleass RJ, Areschoug T, Lindahl G, Woof JM (2001) Streptococcal IgA-binding proteins bind in the Calpha 2-Calpha 3 interdomain region and inhibit binding of IgA to human CD89. J Biol Chem 276: 8197–8204. 11096107 10.1074/jbc.M009396200

[15] Wines BD, Willoughby N, Fraser JD, Hogarth PM (2006) A competitive mechanism for staphylococcal toxin SSL7 inhibiting the leukocyte IgA receptor, Fc alphaRI, is revealed by SSL7 binding at the C alpha2/C alpha3 interface of IgA. J Biol Chem 281: 1389–1393. 16293625 10.1074/jbc.M509334200

[16] Batten MR, Senior BW, Kilian M, Woof JM (2003) Amino acid sequence requirements in the hinge of human immunoglobulin A1 (IgA1) for cleavage by streptococcal IgA1 proteases. Infect Immun 71: 1462–1469. 12595464 10.1128/IAI.71.3.1462-1469.2003 PMC148859

[17] Senior BW, Woof JM (2005) Effect of mutations in the human immunoglobulin A1 (IgA1) hinge on its susceptibility to cleavage by diverse bacterial IgA1 proteases. Infect Immun 73: 1515–1522. 15731049 10.1128/IAI.73.3.1515-1522.2005 PMC1064975

[18] Chintalacharuvu KR, Chuang PD, Dragoman A, Fernandez CZ, Qiu J, et al (2003) Cleavage of the human immunoglobulin A1 (IgA1) hinge region by IgA1 proteases requires structures in the Fc region of IgA. Infect Immun 71: 2563–2570. 12704129 10.1128/IAI.71.5.2563-2570.2003 PMC153282

[19] Senior BW, Woof JM (2006) Sites in the CH3 domain of human IgA1 that influence sensitivity to bacterial IgA1 proteases. J Immunol 177: 3913–3919. 16951354 10.4049/jimmunol.177.6.3913

[20] Mantis NJ, Rol N, Corthesy B (2011) Secretory IgA’s complex roles in immunity and mucosal homeostasis in the gut. Mucosal Immunol 4: 603–611. 21975936 10.1038/mi.2011.41 PMC3774538

[21] Esteves PJ, Alves PC, Ferrand N, van der Loo W (2002) Hotspot variation at the CH2–CH3 interface of leporid IgG antibodies (Oryctolagus, Sylvilagus and Lepus). Eur J Immunogenet 29: 529–535. 12437613 10.1046/j.1365-2370.2002.00355.x

[22] Esteves PJ, Carmo C, Godinho R, van der Loo W (2006) Genetic diversity at the hinge region of the unique immunoglobulin heavy gamma (IGHG) gene in leporids (Oryctolagus, Sylvilagus and Lepus). Int J Immunogenet 33: 171–177. 16712647 10.1111/j.1744-313X.2006.00588.x

[23] Yang Z (1997) PAML: a program package for phylogenetic analysis by maximum likelihood. Comput Appl Biosci 13: 555–556. 9367129 10.1093/bioinformatics/13.5.555

[24] Yang Z (2007) PAML 4: phylogenetic analysis by maximum likelihood. Mol Biol Evol 24: 1586–1591. 17483113 10.1093/molbev/msm088

[25] Chintalacharuvu KR, Raines M, Morrison SL (1994) Divergence of human alpha-chain constant region gene sequences. A novel recombinant alpha 2 gene. J Immunol 152: 5299–5304. 8189047

[26] Lefranc MP, Pommie C, Kaas Q, Duprat E, Bosc N, et al (2005) IMGT unique numbering for immunoglobulin and T cell receptor constant domains and Ig superfamily C-like domains. Dev Comp Immunol 29: 185–203. 15572068 10.1016/j.dci.2004.07.003

[27] Thompson JD, Higgins DG, Gibson TJ (1994) CLUSTAL W: improving the sensitivity of progressive multiple sequence alignment through sequence weighting, position-specific gap penalties and weight matrix choice. Nucleic Acids Res. 22(22): 4673–80. 10.1093/nar/22.22.4673 PMC308517 7984417

[28] Hall TA (1999) BioEdit: a user-friendly biological sequence alignment editor and analysis program for Windows 95/98/NT. Nucleic Acids Symp Ser (Oxf). 41: 95–98.

[29] Edgar RC (2004) MUSCLE: multiple sequence alignment with high accuracy and high throughput. Nucleic Acids Res. 32(5): 1792–7. 10.1093/nar/gkh340 PMC390337 15034147

[30] Wlasiuk G, Nachman MW (2010) Adaptation and constraint at Toll-like receptors in primates. Mol Biol Evol 27: 2172–2186. 20410160 10.1093/molbev/msq104 PMC3107592

[31] Nielsen R, Yang Z (1998) Likelihood models for detecting positively selected amino acid sites and applications to the HIV-1 envelope gene. Genetics 148: 929–936. 9539414 10.1093/genetics/148.3.929 PMC1460041

[32] Yang Z, Swanson WJ, Vacquier VD (2000) Maximum-likelihood analysis of molecular adaptation in abalone sperm lysin reveals variable selective pressures among lineages and sites. Mol Biol Evol 17: 1446–1455. 11018152 10.1093/oxfordjournals.molbev.a026245

[33] Yang Z, Wong WS, Nielsen R (2005) Bayes empirical bayes inference of amino acid sites under positive selection. Mol Biol Evol 22: 1107–1118. 15689528 10.1093/molbev/msi097

[34] Tamura K, Peterson D, Peterson N, Stecher G, Nei M, et al (2011) MEGA5: molecular evolutionary genetics analysis using maximum likelihood, evolutionary distance, and maximum parsimony methods. Mol Biol Evol 28: 2731–2739. 21546353 10.1093/molbev/msr121 PMC3203626

[35] Pond SL, Frost SD (2005) Datamonkey: rapid detection of selective pressure on individual sites of codon alignments. Bioinformatics 21: 2531–2533. 15713735 10.1093/bioinformatics/bti320

[36] Anisimova M, Nielsen R, Yang Z (2003) Effect of recombination on the accuracy of the likelihood method for detecting positive selection at amino acid sites. Genetics 164: 1229–1236. 12871927 10.1093/genetics/164.3.1229 PMC1462615

[37] Scheffler K, Martin DP, Seoighe C (2006) Robust inference of positive selection from recombining coding sequences. Bioinformatics 22: 2493–2499. 16895925 10.1093/bioinformatics/btl427

[38] Shriner D, Nickle DC, Jensen MA, Mullins JI (2003) Potential impact of recombination on sitewise approaches for detecting positive natural selection. Genet Res 81: 115–121. 12872913 10.1017/s0016672303006128

[39] Kosakovsky Pond SL, Posada D, Gravenor MB, Woelk CH, Frost SD (2006) Automated phylogenetic detection of recombination using a genetic algorithm. Mol Biol Evol 23: 1891–1901. 16818476 10.1093/molbev/msl051

[40] Boehm MK, Woof JM, Kerr MA, Perkins SJ (1999) The Fab and Fc fragments of IgA1 exhibit a different arrangement from that in IgG: a study by X-ray and neutron solution scattering and homology modelling. J Mol Biol 286: 1421–1447. 10064707 10.1006/jmbi.1998.2556

[41] Ramsland PA, Willoughby N, Trist HM, Farrugia W, Hogarth PM, et al (2007) Structural basis for evasion of IgA immunity by *Staphylococcus aureus* revealed in the complex of SSL7 with Fc of human IgA1. Proc Natl Acad Sci U S A 104: 15051–15056. 17848512 10.1073/pnas.0706028104 PMC1986611

[42] Wang Y, Geer LY, Chappey C, Kans JA, Bryant SH (2000) Cn3D: sequence and structure views for Entrez. Trends Biochem Sci 25: 300–302. 10838572 10.1016/s0968-0004(00)01561-9

[43] Rotkiewicz P (2007) iMol Molecular Visualization Program. Available: http://www.pirx.com/iMol.

[44] Murrell B, Wertheim JO, Moola S, Weighill T, Scheffler K, et al (2012) Detecting individual sites subject to episodic diversifying selection. PLoS Genet 8: e1002764. 22807683 10.1371/journal.pgen.1002764 PMC3395634

[45] Vallender EJ, Lahn BT (2004) Positive selection on the human genome. Hum Mol Genet 13 Spec No 2: R245–254. 10.1093/hmg/ddh253 15358731

[46] Kosiol C, Vinar T, da Fonseca RR, Hubisz MJ, Bustamante CD, et al (2008) Patterns of positive selection in six Mammalian genomes. PLoS Genet 4: e1000144. 18670650 10.1371/journal.pgen.1000144 PMC2483296

[47] Arnold JN, Wormald MR, Sim RB, Rudd PM, Dwek RA (2007) The impact of glycosylation on the biological function and structure of human immunoglobulins. Annu Rev Immunol 25: 21–50. 17029568 10.1146/annurev.immunol.25.022106.141702

[48] Brezski RJ, Jordan RE (2010) Cleavage of IgGs by proteases associated with invasive diseases: an evasion tactic against host immunity? MAbs 2: 212–220. 20400859 10.4161/mabs.2.3.11780 PMC2881249

[49] Johnson TA, Qiu J, Plaut AG, Holyoak T (2009) Active site gating regulates substrate selectivity in a chymotrypsin-like serine protease. The structure of *Haemophilus influenzae* IgA1 protease. J Mol Biol 389: 559–574. 19393662 10.1016/j.jmb.2009.04.041 PMC2720633

[50] Wines BD, Ramsland PA, Trist HM, Gardam S, Brink R, et al (2011) Interaction of human, rat, and mouse immunoglobulin A (IgA) with staphylococcal superantigen-like 7 (SSL7) decoy protein and leukocyte IgA receptor. J Biol Chem 286: 33118–33124. 21784854 10.1074/jbc.M111.272252 PMC3190891

